# Etoposide enhances antitumor efficacy of MDR1-driven oncolytic adenovirus through autoupregulation of the *MDR1* promoter activity

**DOI:** 10.18632/oncotarget.5702

**Published:** 2015-10-16

**Authors:** Bing-Hua Su, Gia-Shing Shieh, Yau-Lin Tseng, Ai-Li Shiau, Chao-Liang Wu

**Affiliations:** ^1^ Department of Biochemistry and Molecular Biology, College of Medicine, National Cheng Kung University, Tainan, Taiwan; ^2^ Department of Urology, Tainan Hospital, Ministry of Health and Welfare, Executive Yuan, Tainan, Taiwan; ^3^ Division of Thoracic Surgery, Department of Surgery, National Cheng Kung University Hospital, College of Medicine, National Cheng Kung University, Tainan, Taiwan; ^4^ Department of Microbiology and Immunology, College of Medicine, National Cheng Kung University, Tainan, Taiwan

**Keywords:** oncolytic adenovirus, E1A, YB-1, MDR1

## Abstract

Conditionally replicating adenoviruses (CRAds), or oncolytic adenoviruses, such as E1B55K-deleted adenovirus, are attractive anticancer agents. However, the therapeutic efficacy of E1B55K-deleted adenovirus for refractory solid tumors has been limited. Environmental stress conditions may induce nuclear accumulation of YB-1, which occurs in multidrug-resistant and adenovirus-infected cancer cells. Overexpression and nuclear localization of YB-1 are associated with poor prognosis and tumor recurrence in various cancers. Nuclear YB-1 transactivates the multidrug resistance 1 (MDR1) genes through the Y-box. Here, we developed a novel E1B55K-deleted adenovirus driven by the *MDR1* promoter, designed Ad5GS3. We tested the feasibility of using YB-1 to transcriptionally regulate Ad5GS3 replication in cancer cells and thereby to enhance antitumor efficacy. We evaluated synergistic antitumor effects of oncolytic virotherapy in combination with chemotherapy. Our results show that adenovirus E1A induced E2F-1 activity to augment YB-1 expression, which shut down host protein synthesis in cancer cells during adenovirus replication. In cancer cells infected with Ad5WS1, an E1B55K-deleted adenovirus driven by the E1 promoter, E1A enhanced YB-1 expression, and then further phosphorylated Akt, which, in turn, triggered nuclear translocation of YB-1. Ad5GS3 in combination with chemotherapeutic agents facilitated nuclear localization of YB-1 and, in turn, upregulated the *MDR1* promoter activity and enhanced Ad5GS3 replication in cancer cells. Thus, E1A, YB-1, and the *MDR1* promoter form a positive feedback loop to promote Ad5GS3 replication in cancer cells, and this regulation can be further augmented when chemotherapeutic agents are added. In the *in vivo* study, Ad5GS3 in combination with etoposide synergistically suppressed tumor growth and prolonged survival in NOD/SCID mice bearing human lung tumor xenografts. More importantly, Ad5GS3 exerted potent oncolytic activity against clinical advanced lung adenocarcinoma, which was associated with elevated levels of nuclear YB-1 and cytoplasmic MDR1 expression in the advanced tumors. Therefore, Ad5GS3 may have therapeutic potential for cancer treatment, especially in combination with chemotherapy. Because YB-1 is expressed in a broad spectrum of cancers, this oncolytic adenovirus may be broadly applicable.

## INTRODUCTION

Conditionally replicating adenoviruses (CRAds) or oncolytic adenoviruses have been exploited as a cancer treatment option because they selectively replicate in and kill cancer cells [1, 2]. E1B55K-deleted adenovirus, such as ONYX-015, has been developed and used in clinical trials as an anticancer agent [3, 4]. The therapeutic efficacy of E1B55K-deleted adenovirus for refractory solid tumors has been limited. The mechanism of tumor-selective replication of E1B55K-deleted adenoviruses, including a possible role of p53, is still unclear. Both adenovirus infection and oncogenic transformation induce similar signaling cascades in eukaryotic cells. Consequently, adenovirus mutants with impaired virus replication potency in normal cells are usually complemented for productive replication by distinct cellular pathways deregulated in cancer cells. It was reported that the ability of tumor cells to provide the host protein shutoff and late viral RNA export functions determines their permissiveness for the replication of E1B55K-deleted adenovirus [5, 6]. To develop novel CRAds with high antitumor efficacy and tumor selectivity, apart from understanding the functions of viral genes, further explorations of host factors and signaling pathways that may determine tumor-selective replication of E1B55K-deleted adenovirus are needed.

YB-1 is a multifunctional protein involved in regulation of both transcription and translation of target gene expression [7, 8]. Overexpression of YB-1 is detected in a variety of human cancers and associated with poor prognosis and cancer recurrence [9–11]. YB-1 plays significant pro-oncogenic roles in malignant transformation, cell invasion, and drug resistance in a wide variety of cancers [10–13]. YB-1 phosphorylation by Akt can activate translation of silent mRNA species [14]. Disruption of Akt-mediated YB-1 phosphorylation inhibits its nuclear translocation [15]. Nuclear YB-1 controls transcription of various genes through the Y-box region on their promoters, such as the human multidrug resistance 1 (MDR1) gene [13, 16]. Certain environmental stress conditions cause nuclear accumulation of YB-1, which occurs in multidrug-resistant and adenovirus-infected cancer cells [13, 17, 18]. Notably, YB-1 facilitates adenovirus E2 gene expression through the E2 late promoter and controls E2 gene activity at later stages of viral infection [18, 19], implying that YB-1 may be involved in determining E1B55K-deleted adenovirus replication.

Various strategies have been exploited to design or modify CRAds aiming at improving their selectivity and antitumor efficacy. First type of CRAds features loss-of-function mutation in the virus genome, which is compensated by mutations in cancer cells but not normal cells. Second type of CRAds is designed for transcriptional targeting to control adenovirus replication by placing the viral gene, usually E1A under the control of a tissue- or tumor-specific promoter. We have previously combined both approaches to construct various E1B55K-deleted adenoviruses under the control of pan-cancer specific promoters [20–23]. These CRAds have potential as broad-spectrum anticancer agents for targeting tumors and cancer stem cells.

In the present study, we sought to construct a new CRAd, designated Ad5GS3, under the transcriptional control of the *MDR1* promoter, which is a Y-box-containing promoter and can be transactivated by YB-1. We demonstrate that E1A, YB-1, and the *MDR1* promoter form a positive feedback loop to promote Ad5GS3 replication in cancer cells, and this regulation can be further augmented when chemotherapeutic agents are added. Therefore, E1B55K-deleted adenoviruses driven by YB-1 responsive promoters, such as the *MDR1* promoter, are promising anticancer agents, particularly in combination with chemotherapy.

## RESULTS

### Adenovirus E1A and Ad5WS1 upregulates YB-1 expression through E2F-1, which is associated with replication of Ad5WS1 in cancer cells

**Figure 1:**
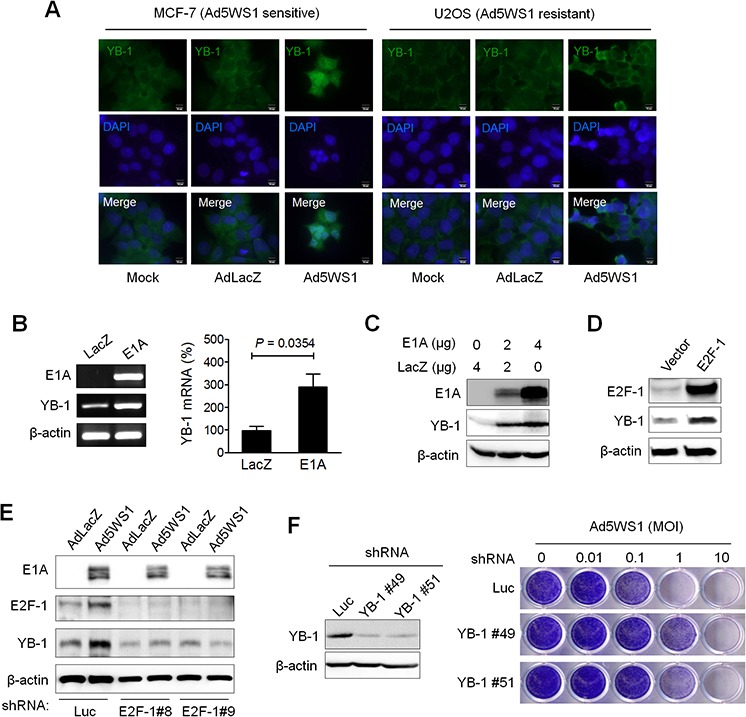
Adenovirus E1A and Ad5WS1 upregulates YB-1 expression, and knockdown of YB-1 decreases Ad5WS1-induced cytolysis in MCF-7 cells **A.** MCF-7 and U2OS cells were infected with AD5WS1, AdLacZ, or mock-infected. After 48 h, the cells were fixed and immunostained with anti-YB-1 antibody followed by fluorescein-conjugated secondary antibody. Nuclei were counterstained with DAPI. Fluorescence signals for YB-1 (green) and the nucleus (blue) were examined by fluorescence microscopy. **B.** MCF-7 cells were transfected with 4 μg of pTCY-E1A or pTCY-LacZ that served as the negative control. After 48 h, the mRNA levels of YB-1, E1A, and β-actin (as quantitative control) were detected by RT-PCR (*left panel*). The YB-1 mRNA level was also quantified by real-time quantitative RT-PCR (*right panel*). Values shown are mean ± SEM (*n* = 3). **C.** MCF-7 cells transfected with pTCY-EIA or pTCY-LacZ, and the expression of E1A, YB-1, and β-actin (as quantitative control) proteins was detected 48 h later by immunoblotting. **D.** MCF-7 cells transfected with pCMV-sport6-E2F-1 or pCDNA3.1 (control vector) were examined 48 h later by immunoblotting. **E.** MCF-7 clones stably overexpressing shE2F-1 or shLuc were infected with Ad5WS1 or AdLacZ at an MOI of 1. After 48 h, cell lysates were analyzed by immunoblotting. **F.** MCF-7 clones stably overexpressing shYB-1 or shLuc were examined for the expression of YB-1 and β-actin by immunoblotting (*left panel*). YB-1 knockdown and control cells were infected with serial 10-fold dilutions of Ad5WS1, and the CPE was monitored by crystal violet staining at 6 days postinfection (*right panel*).

To unravel the role of adenoviral E1A in E1B55K-deleted adenovirus replication, duplicated microarray analysis was performed to determine differential gene expression in MCF-7 cells infected with Ad5WS1 that expressed E1A or Adnull that did not express E1A. We focused on searching for genes with regulatory functions in the translational control, such as RNA binding or transport, importantly involved in E1B55K-deleted adenovirus replication (Supplementary Table S1). Among a total of 10,339 genes showing at least 1.5-fold changes in the expression levels, we chose to study YB-1, which was upregulated in Ad5WS1-infected cells. To confirm the microarray results, Ad5WS1 or AdLacZ was used to infect MCF-7 and U2OS cells known to be sensitive and resistant to oncolytic adenovirus infection, respectively [3]. Expression and localization of YB-1 were observed by fluorescence microscopy. Cytoplasmic and nuclear expression of YB-1 was evident in MCF-7 cells infected with Ad5WS1, but not in those infected with AdLacZ of mock-infected, whereas YB-1 was observed, to a lesser extent, only in the cytoplasm in Ad5WS1-infected U2OS cells (Figure 1A). RT-PCR, quantitative real-time RT-PCR, and immunoblot analyses confirmed overexpression of E1A mRNA and protein in MCF-7 cells transfected with an E1A expression plasmid, which resulted in upregulation of mRNA and protein expression of YB-1 (Figure 1B, 1C). In our microarray data, both YB-1 and E2F-1 were upregulated in Ad5WS1-infected cells. In adenovirus-infected cells, E1A acts to sequester pRB tumor suppressor protein and thereby releases transcriptionally active E2F transcription factor [24, 25]. Figure 1D shows that YB-1 expression was elevated in MCF-7 cells transfected with an E2F-1 expression vector. While Ad5WS1-infected MCF-7 cells expressed higher levels of both E2F-1 and YB-1 compared with AdLacZ-infected cells following transduction of lentiviral vectors expressing shRNA specific to luciferase (Luc), knockdown of E2F-1 with lentiviral vectors expressing shRNA specific to E2F-1 abrogated such effects (Figure 1E), suggesting that E1A upregulates YB-1 expression through E2F-1. Furthermore, knockdown of YB-1 in MCF-7 cells rendered cell more resistant to Ad5WS1-induced cytolysis (Figure 1F).

### Ad5WS1 and wild-type adenoviruses inhibit host protein synthesis through YB-1 in cancer cells

**Figure 2:**
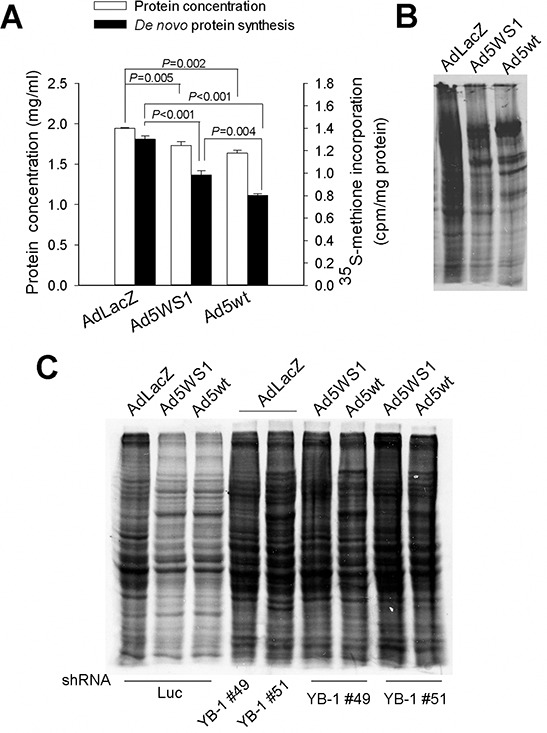
Ad5WS1 and wild-type adenovirus inhibit host protein synthesis through YB-1 in MCF-7 cells **A, B.** The *de novo* protein synthesis in virus-infected cells were examined by pulse-chase analysis (A) and autoradiography (B) MCF-7 cells that had been infected with Ad5WS1, Ad5wt, or AdLacZ at an MOI of 10 for 48 h were starved in cysteine/methionine-free medium for 1 h and then labeled with [^35^S]-Met for 3 h. The concentrations of total proteins were quantified, and [^35^S]-Met labeled *de novo* synthesized proteins were detected by β-counter. Values shown are mean ± SEM (*n* = 3). Normalized protein lysates were separated by gel electrophoresis. The gels were fixed, dried, exposed to X-film for 12 h, and visualized with a phosphorimager. **C.** MCF-7 clones stably overexpressing shYB-1 or shLuc were infected with Ad5WS1, Ad5wt or AdLacZ for autoradiography analysis as described above.

As E1B55K shuts off host cell protein synthesis [5, 6], we evaluated the differences of protein synthesis among MCF-7 cells infected with Ad5WS1, Ad5wt (wild-type adenovirus type 5), and AdLacZ. The inhibition of *de novo* protein synthesis was in the order of Ad5wt > Ad5WS1 > AdLacZ, as revealed by [^35^S]-methionine pulse-chase analysis (Figure 2A). Total protein levels were also lower in cancer cells infected with Ad5wt and Ad5WS1 than AdLacZ (Figure 2A). Given that Ad5wt and Ad5WS1 contain the E1A gene, it seems reasonable to correlate E1A expression with inhibition of protein synthesis. Autoradiography assay confirmed protein synthesis inhibition in Ad5WS1- and Ad5wt-infected cells (Figure 2B). As E1A induced YB-1 overexpression and led to translational suppression, we determined whether adenovirus-induced inhibition of protein synthesis was associated with YB-1 expression. Inhibition of protein synthesis by Ad5WS1 or Ad5wt was abrogated by knockdown of YB-1 expression (Figure 2C). Collectively, these results suggest that E1A induces YB-1 overexpression, thereby inhibiting host protein synthesis during adenovirus replication.

### E1A-induced YB-1 overexpression leads to enhanced Akt phosphorylation

**Figure 3:**
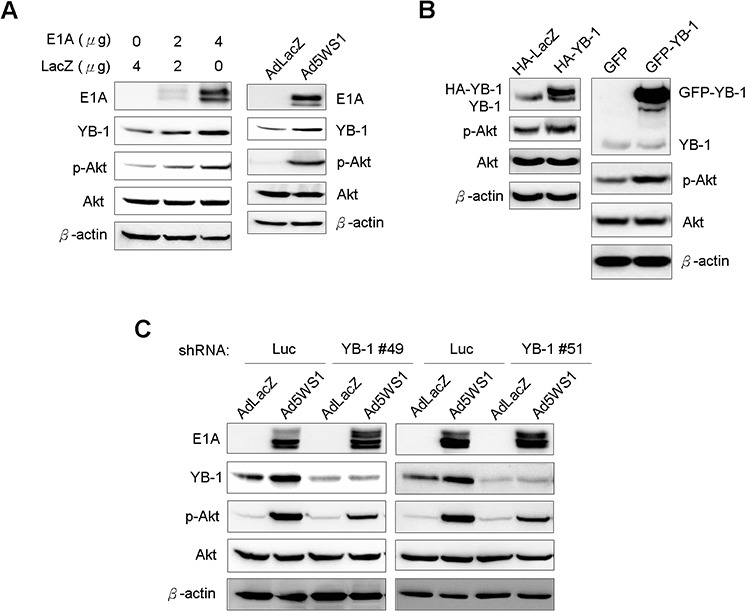
E1A induces YB-1 expression and, in turn, enhances Akt phosphorylation in MCF-7 cells **A.** MCF-7 cells were transfected with 4 μg of pTCY-E1A or TCY-LacZ or infected with Ad5WS1 or AdLacZ at an MOI of 1. **B.** 293T cells were transfected with 4 μg of HA-tagged YB-1, GFP-fused YB-1, or control vectors. **C.** MCF-7 cells stably overexpressing shYB-1 or shLuc were infected with Ad5WS1 or AdLacZ at an MOI of 1. After 48 h, all the cell lysates were examined by immunoblotting (A, B, C).

As inhibition of host protein synthesis activates Akt [26, 27] and YB-1 upregulation enhances Akt activity [28], we next determined whether E1A enhanced phosphorylated Akt (p-Akt) levels in cancer cells. Figure 3A shows YB-1 and p-Akt levels were concomitantly elevated in MCF-7 cells transfected with E1A or infected with Ad5WS1. Overexpression of YB-1 by lentivirus-mediated delivery of HA-tagged YB-1 or GFP-fused YB-1 also enhanced Akt phosphorylation (Figure 3B). Reciprocally, knockdown of YB-1 expression reduced p-Akt levels in Ad5WS1-infected cells (Figure 3C). Taken together, these results suggest that adenoviral E1A enhances Akt phosphorylation through the YB-1 signaling pathway in Ad5WS1-infected cancer cells.

### E1A induces phosphorylation and nuclear localization of YB-1 through p-Akt and thereby enhance cytolytic activity of Ad5WS1

**Figure 4:**
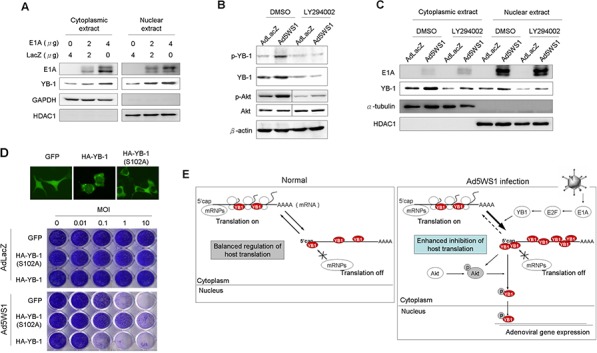
E1A and Ad5WS1 induce phosphorylation and nuclear translocation of YB-1 through p-Akt and thereby enhance cytolytic activity of Ad5WS1 in MCF-7 cells **A.** Cells were transfected with pTCY-E1A and pTCY-LacZ. After 48 h, cytoplasmic and nuclear extracts were examined by immunoblotting. HDAC1 and GAPDH served as the nuclear and cytoplasmic markers, respectively. **B, C.** Cells were infected with Ad5WS1 or AdLacZ at an MOI of 1. After 42 h, the cells were treated with the PI3K inhibitor LY294002 (10 μM) for 6 h. Total cell lysates (B) as well as cytoplasmic and nuclear extracts (C) were examined by immunoblotting. HDAC1 and α-tubulin served as the nuclear and cytoplasmic markers, respectively. **D.** MCF-7 clones stably overexpressing HA-tagged YB-1, HA-tagged YB-1(S102A), or GFP were infected with serial 10-fold dilutions of Ad5WS1. After 6 days, CPE was visualized by crystal violet staining (*bottom panel*). Uninfected cells transfected with GFP-fused YB-1, GFP-fused YB-1(S102A), or control vector were examined by fluorescence microscopy. Note that YB-1(S102A) was incapable of nuclear localization (*top panel*). **E.** A proposed model of Ad5WS1replication at early stages of viral infection in permissive cancer cells. E1A expression in cancer cells infected with E1B55K-deleted adenovirus activates E2F-1 expression, and, in turn, induces YB-1 overexpression and inhibits host protein translation, resulting in activating Akt phosphorylation. Reciprocally, Akt phosphorylation also facilitates the phosphorylation of YB-1, leading to YB-1 nuclear localization. Thus, E1A, YB-1, and Akt form a signal loop that can enhance replication of E1B55K-deleted adenovirus in a positive feedback manner.

Because YB-1 phosphorylation is required for its translocation from the cytoplasm into the nucleus to regulate various gene expression [15], we evaluated whether E1A phosphorylated YB-1 and enhanced its nuclear translocation. Figure 4A shows that cytoplasmic and nuclear levels of YB-1 in MCF-7 cells were increased after transfection with E1A. In Ad5WS1-infected cells, treatment with LY294002, a phosphoinositide 3-kinase (PI3K) inhibitor, abrogated Akt phosphorylation as well as YB-1 expression and phosphorylation (Figure 4B). Notably, levels of nuclear YB-1 were also decreased in Ad5WS1-infected cells treated with the PI3K inhibitor (Figure 4C). An approximately 10-fold increase in the cytopathic effect (CPE) was observed in cells overexpressing wild-type YB-1 compared with those overexpressing GFP (Figure 4D). However, cells overexpressing YB-1(S102A), which was incapable of nuclear translocation due to mutation at the Akt phosphorylation site [15, 29], exhibited approximately 100-fold more resistant to Ad5WS1 infection than those overexpressing wild-type YB-1 (Figure 4D). As expected, AdLacZ did not induce any CPE in the three stably overexpressing cells. These results suggest that Akt phosphorylation facilitated nuclear translocation of YB-1 to enhance Ad5WS1-induced CPE. By fluorescence microscopy, we also confirmed that the mutant YB-1(S102A) failed to translocate into the nucleus, whereas wild-type YB-1 was expressed both in the nucleus and cytoplasm in the uninfected cells (Figure 4D). Therefore, we conclude that E1A can upregulate YB-1 expression to phosphorylate Akt, and p-Akt, in turn, can also induce YB-1 phosphorylation and nuclear translocation, resulting in promoting E1B55K-deleted adenovirus cytolysis in cancer cells. Thus, E1A, YB-1, and Akt form a signal loop that can enhance replication of E1B55K-deleted adenovirus in a positive feedback manner (Figure 4E). As a result, viral replication is increases and thereby its cytolytic activity against cancer cells is enhanced.

### Ad5GS3 expresses higher E1A levels and induces higher levels of YB-1 expression and nuclear translocation than Ad5WS1

**Figure 5:**
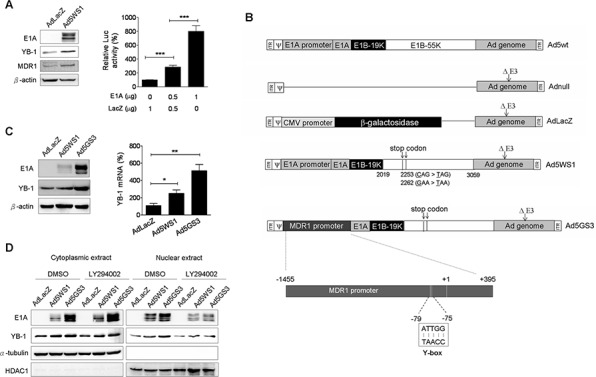
Ad5GS3 is superior to Ad5WS1 in enhancing the expression and nuclear translocation of YB-1 in MCF-7 cells **A.** Cells were infected with Ad5WS1 or AdLacZ at an MOI of 1, and total cell lysates were examined 48 h later by immunoblotting (*left panel*). Cells were cotransfected with pGL3-Basic-MDR1 (0.5 μg) and pTCY-E1A (0.5 or 1 μg). The total amount of plasmid DNA for transfection was kept constant by the addition of pTCY-LacZ. Cells were assessed for MDR1 promoter activities by a luciferase reporter assay (corrected for protein concentration). Values shown are mean ± SEM (*n* = 3). (*right panel*). **B.** Genomic structures of wild-type adenovirus type 5 (Ad5wt) and other adenoviral vectors used in this study. Ad5WS1 (similar to ONYX-015) and Ad5GS3 are replication-competent oncolytic adenoviruses driven by the E1A and *MDR1* promoters, respectively. In Ad5WS1 and Ad5GS3, E1B55K gene (nucleotide 2019–3509 of human adenovirus type 5) carried two point mutations at bases 2253 (C → T) and 2262 (G → T) that generated premature translation stop codons, resulting in the production of a truncated 78-amino acid E1B55K protein. Numbers indicate nucleotide positions of human adenovirus type 5. AdLacZ is an E1-deleted, replication-defective adenoviral vector expressing β-galactosidase driven by the CMV promoter. Adnull is an E1-deleted, replication-defective adenoviral vector expressing no transgene. All of the adenoviral vectors used here contain a deletion in the E3 gene. ITR, inverted terminal repeat;ψ, packaging signal. **C.** Cells infected with adenoviruses at an MOI of 1 were examined 48 h later by immunoblotting (*left panel*). The YB-1 mRNA levels were also detected by real-time quantitative RT-PCR. Values shown are mean ± SEM (*n* = 3) (*right panel*). **D.** Cells infected with Ad5GS3 induced higher nuclear YB-1 and E1A expressions, which were abolished by LY294002 treatment. Cells were infected with Ad5GS3, Ad5WS1, or AdLacZ at an MOI of 1. After 42 h, the cells were treated with LY294002 (10 μM) for 6 h. Cytoplasmic and nuclear extracts were examined by immunoblotting. HDAC1 and α-tubulin served as the nuclear and cytoplasmic markers, respectively.

Because nuclear localization of YB-1 activates MDR1expression in different types of tumors [17, 30, 31], we investigated whether Ad5WS1 induced MDR1 expression in MCF-7 cells. The Ad5WS1-infected cells, as expected, expressed high levels of E1A with concomitant increases in the expression of YB-1 and MDR1 (Figure 5A). Accordingly, expression of E1A upregulated the MDR1 promoter activity in MCF-7 cells (Figure 5A). Therefore, we hypothesized that E1A-induced transactivation of the MDR1 promoter was mediated through the YB-1 activity. We then constructed an E1B55K-deleted adenovirus driven by the MDR1 promoter, designated Ad5GS3 (Figure 5B). Ad5WS3 induced higher levels of E1A and YB-1 proteins and mRNA than did Ad5WS1 (Figure 5C). Compared with Ad5WS1, Ad5GS3 induced higher cytoplasmic and nuclear levels of E1A and YB-1, in which nuclear expression was significantly abrogated by treatment with LY294002 (Figure 5D). Collectively, these results suggest that E1A overexpression, which was driven by the MDR1 promoter in the context of Ad5GS3, exerted a positive feedback regulation in adenovirus replication by enhancing YB-1 nuclear localization to transactivate the MDR1 promoter and thereby to enhance E1A expression. More importantly, this positive feedback loop enhanced adenoviral replication to promote the oncolytic efficacy of Ad5GS3 in cancer cells.

### Ad5GS3 and etoposide collaborates to enhance YB-1 nuclear localization in cancer cells

**Figure 6:**
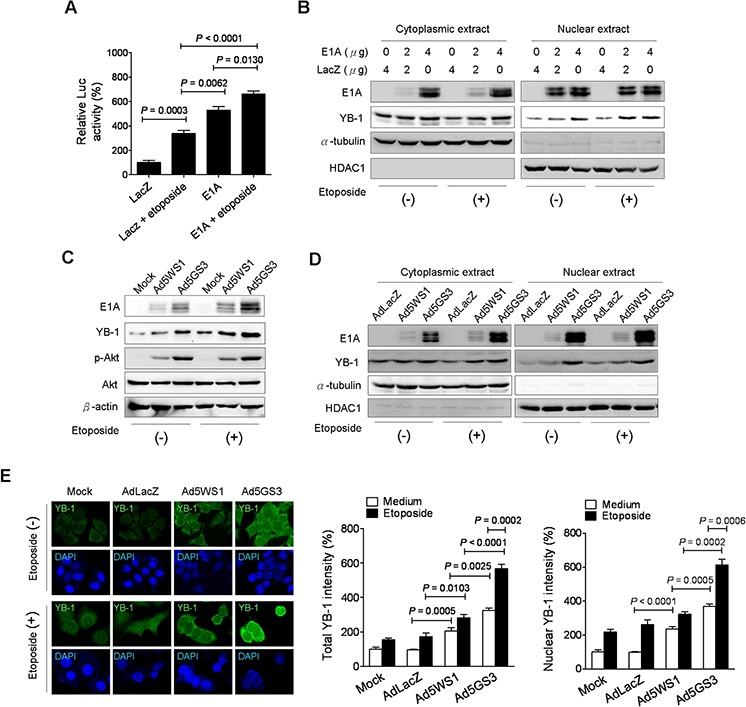
Ad5GS3 and etoposide synergistically enhance nuclear translocation of YB-1 in MCF-7 cells **A.** Cells were cotransfected with pGL3-Basic-MDR1 (0.5 μg) and pTCY-E1A or pTCY-LacZ (1 μg) and treated with or without etoposide (0.5 μg/ml) 24 h later. Cells were assessed for the *MDR1* promoter activities by a luciferase reporter assay (corrected for protein concentration). Values shown are mean ± SEM (*n* = 4). **B.** Cells were transfected with indicated concentrations of pTCY-E1A and pTCY-LacZ. After 6 h, the medium was replaced by fresh medium with or without etoposide (0.5 μg/ml). Cytoplasmic and nuclear extracts were harvested at 48 h posttransfection for immunoblot analysis. **C, D.** Cells were infected with Ad5GS3 or Ad5WS1 at an MOI of 1 with or without etoposide (0.5 μg/ml). Total cell lysates (C) as well as cytoplasmic and nuclear extracts (D) were examined 48 h later by immunoblotting. HDAC1 and α-tubulin served as the nuclear and cytoplasmic markers, respectively. **E.** Cells were infected with adenoviruses at an MOI of 1 with or without etoposide (0.5 μg/ml). After 48 h, they were immunostained with anti-YB-1 antibody followed by fluorescein-conjugated secondary antibody. Nuclei were counterstained with DAPI. The fluorescence signal was examined with confocal microscopy (*left panel*). Fluorescent densities of total YB-1 (*middle panel*) and nuclear YB-1 (*right panel*) were quantified. Values shown are mean ± SEM (*n* = 3).

As MDR1 is activated at the transcriptional level by various chemotherapeutic agents [32, 33], we examined whether E1A combined with etoposide augmented MDR1 promoter activity in MCF-7 cells. As shown in Figure 6A, the MDR1 promoter activity was highest in the cells transfected with E1A in combination with etoposide treatment among the four groups. Moreover, E1A alone induced higher MDR-1 promoter activity than did etoposide. Figure 6B shows that E1A combined with etoposide induced higher levels of nuclear YB-1 expression than either treatment alone. Infection with Ad5WS1 and, in particular, Ad5GS3 enhanced the expression of E1A, YB-1, and p-Akt, and these enhancing effects were further augmented when combined with etoposide (Figure 6C). Levels of nuclear and cytoplasmic YB-1 in MCF-7 cells were in the order: Ad5GS3 plus etoposide > Ad5GS3 alone > Ad5WS1 plus etoposide > Ad5WS1 alone > etoposide alone or AdLacZ alone (Figure 6D). Confocal fluorescence imaging and quantification of the fluorescent density of total and nuclear YB-1 also revealed that highest intensity of nuclear and cytoplasmic YB-1 was observed in cells treated with Ad5GS3 in combination with etoposide (Figure 6E). Therefore, in Ad5GS3-infected cancer cells, etoposide upregulates YB-1 expression as well as p-Akt activity, resulting in YB-1 translocation into the nucleus to transactivate the MDR1 promoter and consequently to overexpress E1A, thereby facilitating Ad5GS3 replication. The strategy of combination therapy with Ad5GS3 and etoposide provides a positive correlation between etoposide and Ad5GS3 replication.

### Defects in the expression and nuclear translocation of YB-1 restrict Ad5GS3 replication in cancer cells

**Figure 7:**
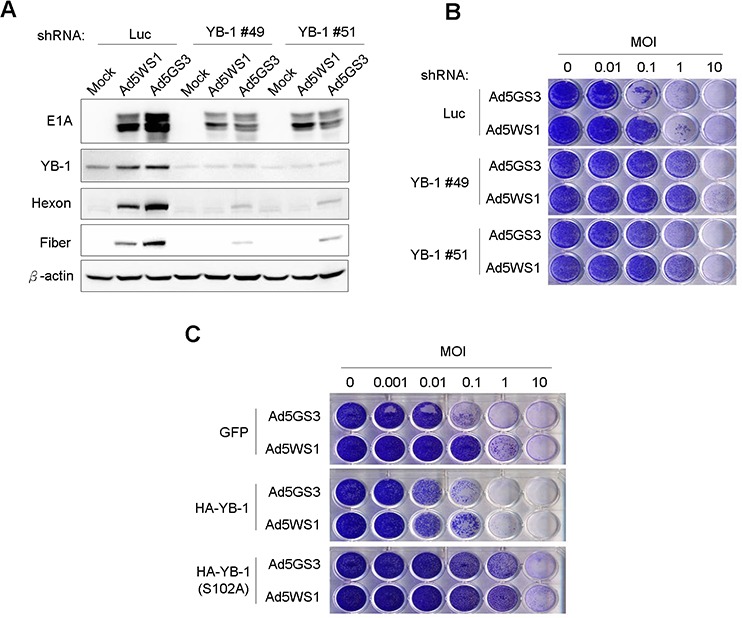
Knockdown of YB-1 decreases, whereas overexpression of YB-1 increases the cytolytic activity of Ad5GS3 and Ad5WS1 against MCF-7 cells **A.** MCF-7 clones stably expressing shYB-1 or shLuc were infected with Ad5GS3, Ad5WS1, or AdLacZ at an MOI of 1. After 48 h, total cell lysates were examined by immunoblotting. **B.** MCF-7 clones stably expressing shYB-1 or shLuc were infected with serial 10-fold dilutions of Ad5GS3 or Ad5WS1. After 6 days, CPE was visualized by crystal violet staining. **C.** MCF-7 clones stably overexpressing HA-tagged YB-1, HA-tagged YB-1(S102A), or GFP were infected with serial 10-fold dilutions of Ad5GS3 or Ad5WS1. After 6 days, CPE was visualized by crystal violet staining.

Next, we studied whether YB-1 dominated the replication of Ad5GS3. As shown in Figure 7A, E1A and YB-1, as well as hexon and fiber proteins, which are two adenoviral late proteins representative of productive adenoviral replication, were detected in MCF-7 cells infected with Ad5GS3 and, to a lesser extent, Ad5WS1. However, knockdown of YB-1 expression diminished the expression of E1A and almost completely abrogated the expression of viral hexon and fiber proteins in the virus-infected cells. These results suggest that YB-1 is crucial for both Ad5WS1 and Ad5GS3 replication in cancer cells. In accordance with these results, knockdown of YB-1 in cancer cells rendered them 10–100-fold more resistant to cytolysis induced by Ad5GS3 or AD5WS1 (Figure 7B). To further confirm these observations, we infected MCF-7 stable clones overexpressing YB-1, YB-1(S102A), or GFP, with Ad5GS3 or Ad5AS1 and tested their susceptibility to viral infection. Both adenoviruses exhibited at least a 10-fold higher cytolytic activity in YB-1-overexpressing cells than in GFP-overexpressing cells (Figure 7C). In addition, cells overexpressing YB-1(S102A) even became more resistant to the cytolysis induced by Ad5GS3 or Ad5WS1 than the control cells overexpressing GFP (Figure 7C). Taken together, these results indicate that YB-1 is crucial for the replication and cytolytic activity of Ad5GS3 and Ad5WS1 in cancer cells.

### Ad5GS3 in combination with etoposide synergistically suppresses tumor growth and prolongs survival in A549 tumor-bearing mice

**Figure 8:**
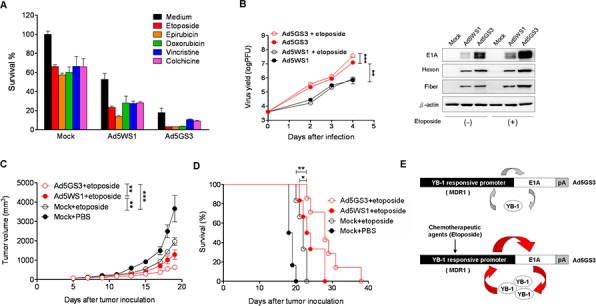
Ad5GS3 synergizes with etoposide to enhance antitumor activity *in vitro* and *in vivo* **A.** A549 lung cancer cells were infected with Ad5GS3 or Ad5WS1 at an MOI of 0.1 for 6 h followed by treatment with etoposide (0.5 μg/ml), epirubicin (0.5 μg/ml), doxorubicin (0.1 μg/ml), vincristine (0.1 μg/ml), and colchcine (0.02 μg/ml). After 6 days, cell survival was determined with the MTT assay. Values shown are mean ± SEM (*n* = 4). **B.** A549 cells were infected with Ad5GS3 or Ad5WS1 and treated with or without etoposide as described in A. The cells and culture supernatants were harvested at indicated time points. Virus yields were quantified by the plaque assay (*left panel*) and confirmed by immunoblotting for detecting the expression of adenovirus late proteins (hexon and fiber) (*right panel*). Values shown are mean ± SEM (*n* = 4). **C, D.** Male NOD/SCID mice at 6 to10 weeks of age that had been inoculated subcutaneously with A549 cells (10^7^) at day 0 were intraperitoneally treated with etoposide (2 mg/kg) daily from day 5 to day 8 alone or in combination with intratumoral injection of Ad5GS3 or Ad5WS1 (10^8^ PFU) at day 5. Tumor volumes (mean ± SEM; *n* = 6) (C) and Kaplan-Meier survival curves (D) are shown. *, *P* < 0.05; **, *P* < 0.01; ***, *P* < 0.001. **E.** A proposed model for the positive feedback loop of E1A, YB-1, and the *MDR1* promoter to regulate Ad5GS3 replication in cancer cells, especially in combination with chemotherapeutic agents. In tumor cells infected with Ad5GS3, expression of E1A is controlled by the *MDR1* promoter, which is frequently active in tumor cells. E1A, in turn, induces YB-1 expression and nuclear translocation, resulting in transactivating the *MDR1* promoter. There is a positive feedback loop between E1A, nuclear YB-1, and the *MDR1* promoter in Ad5GS-sensitive tumors (*top*). In the presence of chemotherapeutic agents, such as etoposide, higher E1A is expressed by Ad5GS3 driven by the *MDR1* promoter, which is upregulated by chemotherapeutic agents through elevated expression of nuclear YB-1 in tumor cells infected with Ad5GS3. E1A then induces YB-1 overexpression and nuclear translocation, leading to transactivating the *MDR1* promoter. Therefore, this positive feedback loop would amplify the production of Ad5GS3 and promote its oncolytic activity to a large extent (*bottom*).

To evaluate the tumor-specific oncolytic efficacy of Ad5GS3, we compared the MDR1 promoter activities in a panel of human cancer and normal cells. MDR1 promoter activity was much higher in cancer cells than in normal cells (HEL299 and Chang liver cells) (Supplementary Figure S1A). Etoposide and epirubicin upregulated the MDR1 promoter activity in cancer cells (Supplementary Figure S1B, S1C), but not in their normal cell counterparts (Supplementary Figure S1D). Thus, following etoposide treatment, the differential MDR1 promoter activity between cancer and normal cells was exploited for therapeutic intervention for cancers. In the promoter assays with or without etoposide exposure, the most significant difference in the MDR1 promoter activity between cancer and normal cells was noted in A549 cells. Thus, we chose A549 cells to evaluate the antitumor activity of Ad5GS3 *in vitro* and *in vivo*. Ad5GS3 exerted higher cytolytic activity than Ad5WS1 in A549 cells when treated alone (Figure 8A). Various chemotherapeutic agents, including etoposide, epirubicin, doxorubicin, vinblastine, and chochicine have been reported to activate the MDR1 promoter [33]. We therefore used these drugs in combination with Ad5GS3 or Ad5WS1 to test their potential synergistic antitumor effects. Ad5GS3 combined with either one of the tested drugs displayed much higher cytolytic activity than Ad5WS1 combined with the respective drugs (Figure 8A). The combination effects of oncolytic adenoviruses plus chemotherapeutic agents were evaluated for the cytotoxicity of A549 cells using the coefficient of drug interaction (CDI). The values of the CDI for any combination were less than 1, indicative of a synergistic effect (Table 1). Ad5GS3 in combination with etoposide displayed the best synergistic cytolytic effect against A549 lung cancer cells. Accordingly, virus yield was the highest over time in A549 cells treated with Ad5GS3 plus etoposide compared with those treated with Ad5GS3 alone, Ad5WS1, or Ad5WS1 plus etoposide (Figure 8B). These results were also verified by detecting the expression of viral hexon and fiber proteins in the treated cells (Figure 8B). In contrast, Ad5GS3 and Ad5WS1 had only negligible effects on cell survival in HEL299 cells (human lung fibroblasts) regardless of etoposide or epirubicin treatment (Supplementary Figure S2).

**Table 1:** The CDI values of Ad5GS3 and Ad5WS1 in combination with various chemotherapeutic drugs

	Coefficient of drug interaction (CDI)				
	Etoposide	Epirubicin	Doxorubicin	Vincristine	Colchicine
Ad5GS3	0.27	0.31	0.33	0.89	0.78
Ad5WS1	0.67	0.46	0.88	0.79	0.81

On the basis of our *in vitro* data, we evaluated the antitumor effects of Ad5GS3 combined with etoposide in A549 tumor-bearing NOD/SCID mice. Remarkably, Ad5GS3 plus etoposide synergistically suppressed tumor growth (Figure 8C) and prolonged survival (Figure 8D) in tumor-bearing mice treated with either Ad5WS1 plus etoposide (*P* < 0.05 for tumor volume and survival) or etoposide alone (*P* < 0.0001 for tumor volume and *P* < 0.05 for survival). Thus, our *in vitro* and animal studies demonstrate that Ad5GS3 in combination with etoposide exhibited the most potent antitumor activity against A549 lung tumors. Our working model is shown in Figure 8E.

### Nuclear translocation of YB-1 predisposes clinical lung adenocarcinoma cells to cytolysis induced by Ad5GS3

**Figure 9:**
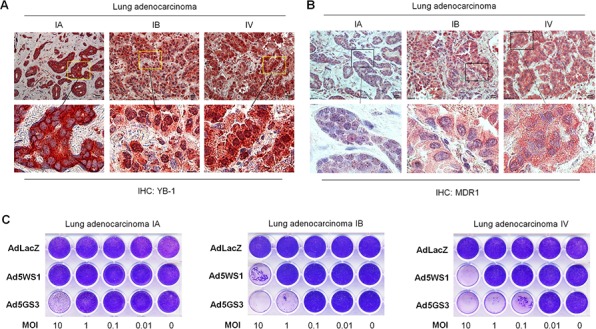
Correlation of cytolytic activity of Ad5GS3 with YB-1 nuclear expression and MDR1 expression in clinical lung adenocarcinoma **A, B.** Expression of YB-1 (A) and MDR1 (B) in human lung adenocarcinoma tissue of varying stages was detected by immunohistochemical staining. Scale bars shown in upper panels correspond to 20 μm, and the boxed areas in upper panels (original magnification × 400) are magnified and shown in lower panels. **C.** Primary cells (5 × 10^5^) derived from human lung adenocarcinoma tissue were cultured in 24-well plates and infected with serial 10-fold dilutions of Ad5GS3, Ad5WS1, or AdLacZ. After 6 days, CPE was visualized by crystal violet staining.

Finally, we evaluated the oncolytic activity of Ad5GS3 in primary cancer cells obtained from clinical lung adenocarcinoma tissue. We examined the expression of MDR1 as well as the expression and localization of YB-1 in the clinical lung adenocarcinoma tissue. Immunohistochemical staining reveals that nuclear accumulation of YB-1 was more evident in the IV stage than the IA and IB stage tumors (Figure 9A). Furthermore, expression of MDR1, located in the cytoplasm and cell membrane, was higher in the IV stage tumor, as compared with the IA and IB tumors (Figure 9B). The CPE was increased by 10–100 folds induced by Ad5GS3 in the stage IV tumor expressing nuclear YB-1, as compared with that in the stage 1A and 1B tumors expressing cytoplasmic YB-1 (Figure 9C). Furthermore, whereas AdLacZ did not display any cytolytic activity as expected, Ad5GS3 exerted higher cytolytic activity than Ad5WS1 against lung adenocarcinoma cells. Collectively, these results suggest that advanced tumors that express high levels of MDR1 and nuclear YB-1 are susceptible to Ad5GS3-induced cytolysis.

## DISCUSSION

Shutoff of host cell protein synthesis by E1B55K is required for adenovirus replication [34]. In tumor cells infected with E1B55K-deleted adenovirus, host protein synthesis is also shut down for adenoviral replication [5, 6]. It is controversial as to whether E1A shutdown host translation to induce adenovirus replication. In the present study, we show that cytoplasmic and nuclear YB-1 overexpression induced by E1A or Ad5WS1 could shutdown host protein translation. It was also suggested that selective replication of E1B55K-deleted adenoviruses depends on enhanced E1A expression in cancer cells [35]. Previous studies have demonstrated that cytoplasmic localization of YB-1 inhibits protein synthesis [36]. Our data also show that cytoplasmic YB-1 overexpression induced by E1A transfection or E1B55K-deleted adenovirus infection was involved in inhibiting host protein synthesis in cancer cells. E1A targets to the retinoblastoma protein (pRb) to enhance the activity of E2F-1 transcription factor [24, 25]. Our microarray data also reveal that both E2F-1 and YB-1 were upregulated in Ad5WS1-infected breast cancer cells. We show that YB-1 activity was correlated with E2F-1 expression levels in E2F-1-trasfected or E1B55K-deleted adenovirus-infected cells. Furthermore, our data indicate that E1A induced E2F-1 activity and then further facilitated cytoplasmic YB-1 expression, which consequently shut down host protein translation. Thus, these results explain how E1A compensates for the loss of E1B55K protein in inhibiting host protein synthesis at the initial stage of E1B55K-deleted adenovirus replication.

Adenoviral E4 can activate the Akt/mTOR pathway to induce viral replication [37]. Phosphorylated Akt induces the phosphorylation of YB-1 and then further promotes its nuclear accumulation [29, 38], leading to enhancing adenoviral replication [18, 19]. In the present study, we examined whether adenoviral E1A induced Akt phosphorylation to facilitate YB-1 nuclear translocation in breast cancer cells. Our results show that E1A enhanced cytoplasmic YB-1 expression and Akt phosphorylation, and, in turn, induced YB-1 nuclear translocation, which could be blocked by PI3K inhibitors. In adenovirus-infected cancer cells, E1B55K facilitates nuclear accumulation of YB-1, which controls the early to late phase transition during the life cycle of adenovirus [18, 19]. Our results show that Ad5WS1 induced nuclear accumulation of YB-1 in sensitive but not resistant cancer cells. Moreover, overexpression of exogenous YB-1 enhanced Ad5WS1 replication. In contrast, cancer cells incapable of YB-1 expression and nuclear localization became resistant to the replication and cytolysis of E1B-55K-deleted adenovirus. Our findings support a working model that E1A-mediated Akt phosphorylation can promote YB-1 nuclear translocation and, in turn, enhance the replication and antitumor activity of E1B55K-deleted adenovirus. We also demonstrate the association between overexpression of nuclear YB-1 and antitumor activity of E1B-55K-deleted adenovirus in clinical lung adenocarcinoma cells.

E1A driven by tumor-specific promoters has been exploited for constructing oncolytic adenoviruses [20–22, 39]. Our results show that E1A enhanced the expression and nuclear localization of YB-1 in Ad5WS1-sensitive cancer cells, which promoted the antitumor activity of Ad5WS1. Therefore, for the replication of E1B55K-deleted adenovirus in cancer cells, it is ideal that E1A is controlled by a tumor-specific promoter that is regulated by nuclear YB-1. Previous studies have suggested that overexpression and nuclear localization of YB-1 are associated with drug resistance and tumor progression in various cancers [9, 40–42]. Nuclear YB-1 induces MDR1 expression in chemotherapy-resistant cancers [13, 16, 30, 40]. Thus, YB-1 expression is a significantly differential marker between chemoresistant cancer cells and normal cells. In the present study, the *MDR1* promoter driven by nuclear YB-1 provides high tumor-specific regulation of E1A expression and Ad5GS3 replication. Ad5GS3 driven by the *MDR1* promoter exerted higher cytolytic activity to kill cancer cells than Ad5WS1 driven by the E1A promoter, but displayed negligible cytotoxicity in normal cells. The *MDR*1 promoter activity is significantly higher in cancer cells than in normal cells. Wild-type p53 inhibits MDR1 expression through direct interaction with YB-1 [43], whereas mutant p53 strongly upregulates the transcriptional activity of the *MDR1* promoter and expression of the endogenous MDR1 [44]. These findings suggest that Ad5GS3, which is driven by the *MDR1* promoter, augments the therapeutic window between tumor and normal cells. In comparison with Ad5WS1, Ad5GS3 induced higher levels of E1A and p-Akt to enhance cytoplasmic and nuclear YB-1 expression, leading to enhanced adenovirus replication. Our results from cell, animal, and clinical studies indicate that Ad5GS3 exerts significantly higher antitumor activity than Ad5WS1. Furthermore, the oncolytic activity of Ad5GS3 is attenuated due to inactive *MDR1* promoter in normal cells. In normal cells, chemotherapy induces wild-type p53 overexpression to decrease the *MDR1* promoter activity. Thus, Ad5GS3 is a safer and more potent oncolytic agent than ONYX-015 [3] or Ad5WS1 [45].

Because nuclear localization of YB-1, which is frequently detected in cancers treated with chemotherapeutic agents, is important for E1B55K-deleted adenovirus replication, synergy of antitumor activity occurs with combination of oncolytic adenoviruses and chemotherapeutic agents. Our results show that etoposide combined with E1A upregulates nuclear YB-1 expression, and subsequently enhances the *MDR-1* promoter activity. In addition, etoposide induced overexpression of E1A and p-Akt, and then further upregulated cytoplasmic and nuclear YB-1 activities in Ad5GS3- and Ad5WS1-infected cancer cells. Moreover, combination of Ad5GS3 with etoposide exhibited highest antitumor effects in *in vitro* and *in vivo* studies. In combination with etoposide, a significant enhancement of Ad5GS3 replication was noted, which explained the synergistic antitumor effect. We provide a novel rationale for the design of oncolytic adenoviruses combined with chemotherapy for cancer therapy. In the combination therapy, etoposide activates endogenous YB-1 expression, thereby enhancing *MDR1* promoter activity and driving more adenoviral E1A expression.

Transcription regulation of the human *MDR1* gene is complex and depends on several *trans*-acting proteins that bind consensus *cis*-elements [46]. The *MDR1* promoter contains Y-box (CCAAT element) found in the inverse complement ATTGG and located at the −79 to −75 sequence position relative to the +1 transcriptional start site. Induction of the *MDR1* gene by various anticancer drugs or UV irradiation involves the Y-box bound by YB-1 [16]. The copy number of a response element within a promoter may influence the magnitude of the promoter activity. We have shown that reporter constructs carrying six copies of the hypoxia response element (HRE), nine copies of the Oct4 response element (ORE), or their combination conjugated to the CMV minimal promoter confer higher transcriptional activities in response to hypoxia and/or Oct-4, as compared with those carrying lower copies of the HRE and/or ORE [20, 21, 47]. We have also generated E1B55K-deleted adenoviruses by replacing its internal E1A promoter with these artificial promoters [20, 21]. These oncolytic adenoviruses are potent and efficacious for treating hypoxic or Oct-4-overexpressing tumors and may be broadly exploited for treating a wide range of primary and metastatic tumors. Because there is only one Y-box in the *MDR1* promoter, it is conceivable that inclusion of multiple copies of the Y-box may increase the transcriptional activity of the promoter in response to YB-1. We have used the template repeated-PCR technique to obtain multiple copies of the Y-box and constructed two luciferase reporter constructs, pGL3–12 × Y-box-CMVmini and pGL3–42 × Y-box-CMVmini, that consist of 12 and 42 copies of the Y-box ligated to the CMV minimal promoter, respectively. We examined the responsiveness of the Y-box in the context of the reporter constructs to YB-1 transactivation in A549 cells (Supplementary Figure S3A). The reporter construct carrying 42 copies of the Y-box conferred higher YB-1-dependent transactivational activity than that carrying 12 copies of the Y-box (Supplementary Figure S3B). Treatment with etoposide enhanced their activity (Supplementary Figure S3C). However, it is still not clear how many copies of the Y-box are required for maximum transactivational activity of the promoter. Thus, the *MDR1* promoter used to drive Ad5GS3 may be modified to insert multiple copies of the Y-box to enhance tumor-selective viral replication and antitumor efficacy.

In the work described here, we show that E1A and Ad5WS1 enhanced Akt phosphorylation through YB-1. In the early stage of adenovirus infection, the E1A proteins can sequester pRb and release repression of E2F, allowing it to activate genes required for entry into S phase and thus facilitate virus replication in the later stage of the infection. The activation of PI3K/Akt pathway in adenovirus-infected cells occurs subsequent to E1A expression [37]. The PI3K inhibitor LY294002 inhibits Akt phosphorylation and thereby restricts adenovirus replication [37]. Importantly, our results indicate that activation of the PI3K/Akt pathway by adenovirus was E1A-dependent. In addition to the PI3K/Akt pathway, E1A can also associate with the insulin receptor substrate (IRS-1) to increase Akt phosphorylation, which contributes to adenovirus-transformed phenotypes and modulates viral infection in an Akt-dependent manner [48]. Notably, in the early stage of adenovirus infection, short-term E1A expression within 48 h is sufficient to activate PI3K/Akt pathway or inhibit pRb to modulate an environment for virus replication. Our results also show that transient transfection of E1A was able to activate Akt phosphorylation. Akt is activated downstream of PI3K and has multiple targets. Akt signaling not only inactivates several pro-apoptotic factors, but also activates transcription factors that upregulate anti-apoptotic genes [49]. E1A has been shown to induce opposite effects, such as proliferation and apoptosis, or transformation and tumor suppression [50]. E1A can repress the transcription of the *HER-2/neu* gene [51], which has led to the development of E1A gene therapy for cancer [52, 53]. Furthermore, E1A can inhibit oncogenic signaling pathways, including Akt signaling [54]. HER-2/neu activates the Akt pathway [55], and E1A mediates sensitization to apoptosis by inactivation of Akt [56, 57]. Furthermore, the anti-oncogenic activity of E1A is not limited to tumors that overexpress HER2/neu [54]. However, these studies used stable cell clones for long-term overexpression of E1A. This experimental condition of E1A expression in cancer cells was different from our condition of short-term expression of E1A in the infection and replication stages of adenovirus. Although oncogenic mutations drive the cells to proliferate, deregulation of these genes also often results in the induction of apoptosis. Therefore, crosstalk between cell proliferation and cell death promotes a balance between proliferation and apoptosis, which limits the growth and survival of cells with oncogenic mutations [58]. Since the interaction of E1A with a wide range of cellular proteins in multiple signal transduction pathways results in multiple biological activities, it is not surprising that E1A has been related to different or opposite biological processes, such as apoptosis, differentiation, and oncogenesis. However, further studies are needed to elucidate the molecular mechanisms underlying different biological activities of E1A.

Numerous clinical trials have been conducted to evaluate the potential of oncolytic virotherapy [59]. Results from clinical trials have indicated that oncolytic viruses hold promise as anticancer agents. Although CRAds have emerged as anticancer agents, the more precise mechanisms by which they kill tumor cells are still unclear. Moreover, targeting of CRAds to tumors aiming at increasing their efficacy and safety profile has become an important issue for virotherapy. YB-1 has pleiotropic functions in the cytoplasm and nucleus. It is mainly localized in the cytoplasm to interact with cytoplasmic proteins and RNA. However, chemotherapy can initiate nuclear translocation of YB-1. In clinical studies of YB-1 expression, nuclear or cytoplasmic localization of YB-1 is closely associated with the expression of MDR1 in various cancers [7]. Nuclear expression of YB-1 is a novel marker of cancer progression. Recent studies also identified a crucial role for YB-1 to induce epithelial-to-mesenchymal transition (EMT), which has a key role in cancer invasion and metastasis [10, 12]. YB-1 also plays an important role in transactivating genes associated with a cancer stem cell phenotype [11, 60], implying that the importance of YB-1 in cancer progression. In the work described here, our results reveal that Ad5GS3 exerted much higher oncolytic activity against stage IV lung adenocarcinoma than stages IA and IB lung adenocarcinoma, which was associated with higher levels of nuclear YB-1 and cytoplasmic MDR1 expression in the late stage tumor. These results highlight the importance of nuclear YB-1 expression in the cytolytic activity of oncolytic adenoviruses and provide the therapeutic potential of Ad5GS3 for clinical cancer therapy. Furthermore, our results provide new insights into the design of a novel oncolytic adenovirus driven by the *MDR-1* promoter, which is regulated by YB-1 that is highly expressed in various cancers, in particular more advanced and chemotherapy-resistant tumors. Ad5GS3 in combination with chemotherapy facilitates nuclear localization of YB-1 and, in turn, upregulates the *MDR1* promoter activity and enhances replication of Ad5GS3 in cancer cells. More importantly, this positive feedback regulation that involves E1A, YB-1, and the *MDR1* promoter to augment Ad5GS3 replication is clinically relevant because of the high incidence of MDR1-resistant tumors.

In conclusion, our work indicates that Ad5GS3 may have therapeutic potential for cancer treatment. More importantly, our results suggest that advanced tumors are good target for Ad5GS3 treatment, especially in combination with chemotherapy. Because YB-1 is expressed in a broad spectrum of cancers, this oncolytic adenovirus may be broadly applicable.

## MATERIALS AND METHODS

### Cell lines, mice, clinical samples, and chemotherapeutic agents

Human breast cancer (MCF-7), lung cancer (A549), osteosarcoma (U2OS), and embryonic kidney cells (293 and 293T) were cultured in Dulbecco's modified Eagle's medium (DMEM) supplemented with 10% fetal bovine serum, 2 mM L-glutamine, and 50 μg/ml gentamicin. NOD.CB17-*Prkdc^scid^* (NOD/SCID) mice were obtained from the Laboratory Animal Center of the National Cheng Kung University (NCKU). The experimental protocol was approved by the Laboratory Animal Care and Use Committee of the NCKU. Clinical specimens were collected from patients with lung adenocarcinoma at the Thoracic Division, Department of Surgery, NCKU Hospital. Informed consent was obtained from all subjects, and the experimental protocol was approved by the Human Experiment and Ethics Committee, NCKU Hospital. Etoposide (Bristol-Myers Squibb, Princeton, NJ), epirubicin (Pfizer Italia Srl, Nerviano, Italy), doxorubicin (Pfizer Italia Srl, Nerviano, Italy), vincristine (Eli Lilly and Company, Indianapolis, IN, USA), and colchicine (Sigma-Aldrich, St. Louis, MO, USA) were used as chemotherapeutic agents.

### Construction and generation of recombinant adenoviruses

The 1.8-kb MDR1 promoter region was excised from pGL3-Basic-MDR1 by digestion with *Kpn*I and *Nhe*I and cloned into the *Kpn*I/*Spe*I (*Nhe*I compatible) sites of pMECA [61]. The inserted fragment was excised by *Bam*HI and *Eco*RV digestions and cloned into the large vector-containing fragment isolated from pAd5YS [20] by digestion with *Hin*dIII, filling-in the cohesive end with klenow enzyme and deoxynucleotide triphosphates, and digestion with *Bam*H1, resulting in pAd5YS/MDR1p. The fragment encompassing sequences of the human MDR1 promoter, E1A, E1B19K, and E1B55K that carries two point mutations to generate premature translation stop codons [39, 45] was excised from pAd5YS/MDR1p by *Bsr*GI and *Mfe*I digestions and cloned into the adenoviral shuttle plasmid pShuttle to generate pShuttle-YS/MDR1p. The resulting vector was linearized with *Pme*I and subsequently cotransformed into *Escherichia coli* strain BJ5183 with pAdEasy-1 to generate recombinant adenoviral plasmid pAd5GS3. Recombinant Ad5GS3 adenovirus was generated in 293 cells as described [20]. Adnull adenovirus was generated by transfection of pAdEasy-1 containing all adenovirus type 5 sequences except E1 and E3 genes into 293 cells as previously described [62]. Ad5wt, AdLacZ, and Ad5WS1, which is an E1B55K-deleted adenovirus similar to ONYX-015, have been described [45, 63]. The genomic structures of the viruses used in the present study are shown in Figure 5B.

### Microarray analysis

Total RNA was extracted from MCF-7 cells infected with Ad5WS1 or Adnull, and their cDNA was synthesized and labeled with Cy5. Microarray hybridization was carried out using Phalanx Biotech Spotted Microarray (HOA_005.1).

### Pulse-chase analysis, transfection, luciferase reporter assay, cell viability assay, and plaque assay

Pulse-chase analysis was performed to measure *de novo* protein synthesis in virus-infected cells as previously described [5]. Lipofectamine 2000 (Invitrogen, Carlsbad, CA) was used for plasmid transfection into cells. The MDR1 promoter activity was analyzed as described [64]. Cells were infected with various adenoviruses at different multiplicities of infection (MOI) and monitored for CPE by crystal violet staining. Cell viability was determined with the MTT assay. Viral yields were quantified by the plaque assay on 293 cells.

### Immunoblot, immunofluorescence, and immunohistochemistry analysis

Total cell lysates were extracted and normalized. Nuclear and cytoplasmic extracts were prepared using hypotonic and hypertonic buffers as previously described [65]. Immunoblot analysis was performed with antibodies against YB-1 (Abcam, Cambridge, UK), phospho-serine102-YB-1 (C34A2, Cell Signaling, Beverly, MA), adenoviral E1A (M58, BD Bioscience, San Diego, CA), Akt (Cell Signaling), phospho-serine473-Akt (Cell Signaling), MDR1 (Calbiochem, Darmstadt, Germany), histone deacetylase 1 (HDAC1) (H-11; Santa Cruz Biotechnology, Santa Cruz, CA), transcription factor E2F-1 (E2F1, Santa Cruz), β-actin (Abcam), α-tubulin (B-7, Santa Cruz), and glyceraldehydes-3-phosphate dehydrogenase (GAPDH) (Abcam) as described [20]. For immunofluorescence staining of YB-1, cells were incubated with anti-YB-1 antibody and subsequently incubated with fluorescein-conjugated goat anti-rabbit IgG (KPL, Gaithersburg, MD). Nuclei were counterstained with DAPI. The fluorescence signal was examined under a laser scanning confocal microscope (FV1000, Olympus, Tokyo, Japan), and the fluorescent pixel density was quantified using the MetaMorph software (Universal imaging Corporation, West Chester, PA). Immunohistochemistry was performed using standard methods as previously described [66]. Briefly, 5-μm-thick sections of formalin fixed and paraffin-embedded human lung cancer tissue blocks were prepared. After deparaffinization and rehydration, tissue sections were blocked with bovine serum albumin, followed by incubation with primary antibodies against YB-1 (Abcam) and MDR1 (Santa Cruz). Tissue sections were incubated with primary antibody at 4°C overnight, followed by incubation with horseradish peroxidase (HRP)-conjugated goat anti-mouse or anti-rabbit IgG (Jackson) at room temperature for 2 h. The reactivity was visualized with aminoethyl carbazole (AEC, red color, Zymed) and counterstained with hematoxylin.

### RT-PCR and real-time quantitative RT-PCR analysis

Total RNA isolation, cDNA synthesis, and PCR were performed as previously described [20]. Real-time quantitative RT-PCR was conducted using the SmartCycler (Cepheid, Sunnuvale, CA). The cDNA and primers were mixed with the SYBR premix Ex Taq (TaKaRa, Kyoto, Japan). Relative mRNA expression of YB-1 was determined using the 2^−ΔΔ*C*^_t_ Method, with value obtained by subtracting the Ct value of GAPDH mRNA from the Ct value of the YB-1 mRNA [67]. The primer sequences used include the sense primer 5′-AGAAATGGCCGCCAGTCTTTTG-3′ and the antisense primer 5′-AAGCGAACACATAATATCTGG GTCCC-3′ for E1A, the sense primer 5′-CAAGAAA GTCATCGCAACGAAGGTT-3′ and the antisense primer 5′-GAGGTACCGACGTTGAGGTGGCT-3′ for YB-1, the sense primer 5′-TAAAACGCAGCTCAG TAACAGTCCG-3′ and the antisense primer 5′-TGG AATCCTGTGGCATCCATGAAAC-3′ for β-actin, and the sense primer 5′-ACTTCAACAGCGACACCCACT-3′ and the antisense primer 5′-GCCAAATT CGTTGTC ATACCAG-3′ for GAPDH.

### Lentivirus-mediated gene transfer

The pLKO.1-puro-based lentiviral vectors containing stem-loop cassettes encoding shRNAs specific for human YB-1 (TRCN 0000007949 and 7951), human E2F-1 (TRCN0000039658 and 39659), and luciferase (TRCN 0000072246) were obtained from the National RNAi Core Facility, Academia Sinica, Taiwan. Knockdown efficiencies were assessed by immunoblotting of lysates from cells transfected with the plasmids encoding respective shRNAs. Three pLKO.1-puro-based expression vectors encoding HA-tagged YB-1, YB-1(S102A) in which serine residue 102 was mutated to alanine [29], and GFP were also used to generate recombinant lentiviruses. They were produced by transient transfection of 293T cells with pLKO.1-puro constructs along with the packaging construct psPAX2 and the VSV-G expression construct pMD2G using the calcium phosphate precipitation method as previously described [68].

### Generation of cells overexpressing YB-1 or YB-1 shRNA

For generation of stable clones, MCF-7 cells were incubated with recombinant lentiviruses expressing YB-1 shRNA for 48 h in the presence of 8 μg/ml polybrene (Sigma-Aldrich, St. Louis, MO). Cells were then incubated in the presence of puromycin (2 μg/ml) for 2 weeks. Stable transfectants expressing YB-1 shRNA were tested for suppression of YB-1 expression, and the cell lines with the most efficient knockdown were selected for subsequent experiments. Similarly, MCF-7 cells expressing HA-tagged YB-1 or its mutants were also examined for overexpression of YB-1.

### Drug interaction analysis

To analyze the effect of the drug combination, A549 cells (1 × 10^5^) cultured in 24-well plates were treated with various chemotherapeutic agents, including etoposide (0.5 μg/ml), epirubicin (0.5 μg/ml), doxorubicin (0.1 μg/ml), vincristine (0.1 μg/ml), and colchicine (0.02 μg/ml) plus Ad5GS3 or Ad5WS1 (MOI = 0.1) for 6 days. The cytotoxic effect was assessed by the MTT assay. The CDI was used to analyze the combinations as previously described [69]. The CDI value was calculated by the following formula: CDI = AB/(A × B). AB is the survival rate of the two-drug combination group relative to the control group, and A or B is the survival rate of the single drug group relative to the control group. The CDI values of <1, =1, and >1 indicate that the drugs are synergistic, additive, and antagonistic, respectively.

### Animal studies

NOD/SCID mice were subcutaneously inoculated with A549 cells (10^7^) at day 0. At day 5, visible and palpable tumors developed at all injection sites (range 41 to 118 mm^3^ and mean ± SD 72.80 ± 17.10 mm^3^). Groups of 6 tumor-bearing mice were treated intraperitoneally with etoposide (2 mg/kg) for 4 consecutive days alone starting from day 5 or in combination with intratumoral injection of 10^8^ plaque-forming units (PFU) of Ad5GS3 or Ad5WS1 at day 5. All mice were monitored for tumor growth and survival. Tumor volumes were measured as described previously [20]. Animals were sacrificed when their primary tumor reached 10% of the body weight.

### Statistical analysis

Statistical significance between groups, unless otherwise stated, was assessed with Student's *t* test. Differences in virus yield and tumor volume were compared by two-way analysis of variance (ANOVA) with repeated measures. Survival analysis was performed using the Kaplan-Meier survival curve and log-rank test.

## SUPPLEMENTARY FIGURES AND TABLE




