# Supplementary material for: Etoposide enhances antitumor efficacy of MDR1-driven oncolytic adenovirus through autoupregulation of the MDR1 promoter activity
Source: Oncotarget. 2015 Oct 16;6(35):38308–26. doi: 10.18632/oncotarget.5702 (PMC4742001; doi:10.18632/oncotarget.5702)
Supplement: Supplementary file 1 [file oncotarget-06-38308-s001.pdf]

SUPPLEMENTARY FIGURES AND TABLE

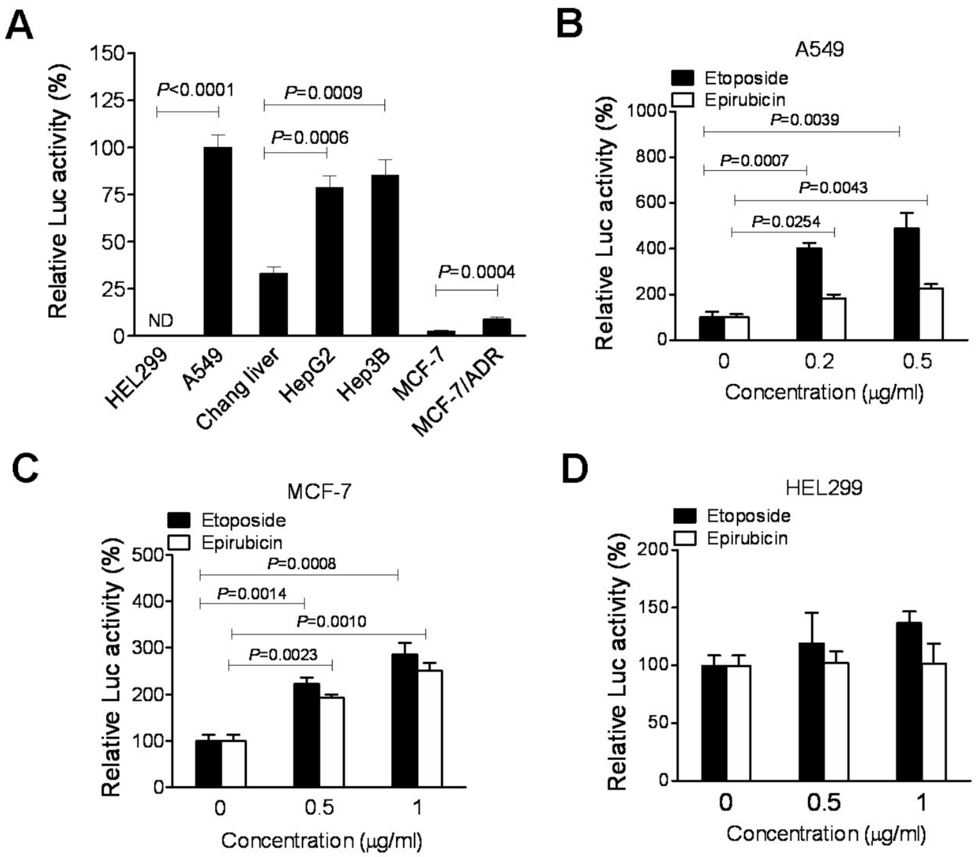

Supplementary Figure S1: Treatment of etoposide and epirubicin enhances the MDR1 promoter activity in cancer cells. A. MDR1 promoter activities in different human cancer and normal cells. Human A549 (lung cancer), MCF-7 (breast cancer), MCF-7/ADR (adriamycin-resistant MCF-7 subline), HepG2 (liver cancer), Hep3B (liver cancer), Chang liver (normal liver), and HEL299 (normal lung fibroblast) cells were cotransfected with the *MDR1* promoter construct pGL3-Basic-MDR1 (0.8 µg) and pTCY-LacZ (0.2 µg). After 48 h, total cell lysates were harvested, and their luciferase activity was determined and normalized with β-galactosidase activity. **B–D.** Etoposide and epirubicin enhances the *MDR1* promoter activity in A549 and MCF-7, but not in HEL299 cells. A549 (B), MCF-7 (C), and HEL299 (D) cells were cotransfected with pGL3-Basic-MDR1 (0.8 µg) and pTCY-LacZ (0.2 µg). After 24 h, cells were treated with various concentrations of etoposide or epirubicin for an additional 24 h. Total cell lysates were harvested, and their luciferase activity was determined and normalized with β-galactosidase activity. Luc, luciferase.

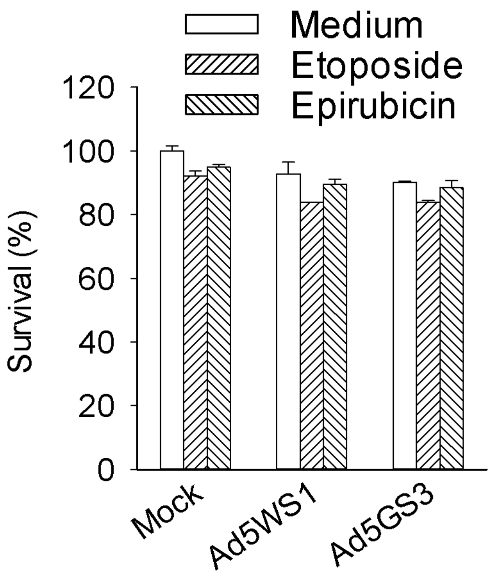

Supplementary Figure S2: Normal HEL299 cells are resistant to cytolysis induced by Ad5GS3 or Ad5WS1. HEL299 cells were infected with Ad5GS3 or Ad5WS1 at an MOI of 0.1. After 6 h, the cells were refed with fresh medium containing etoposide (0.5 µg/ml) or epirubicin (0.5 µg/ml). Cell survival was determined with the MTT assay at 6 days postinfection.

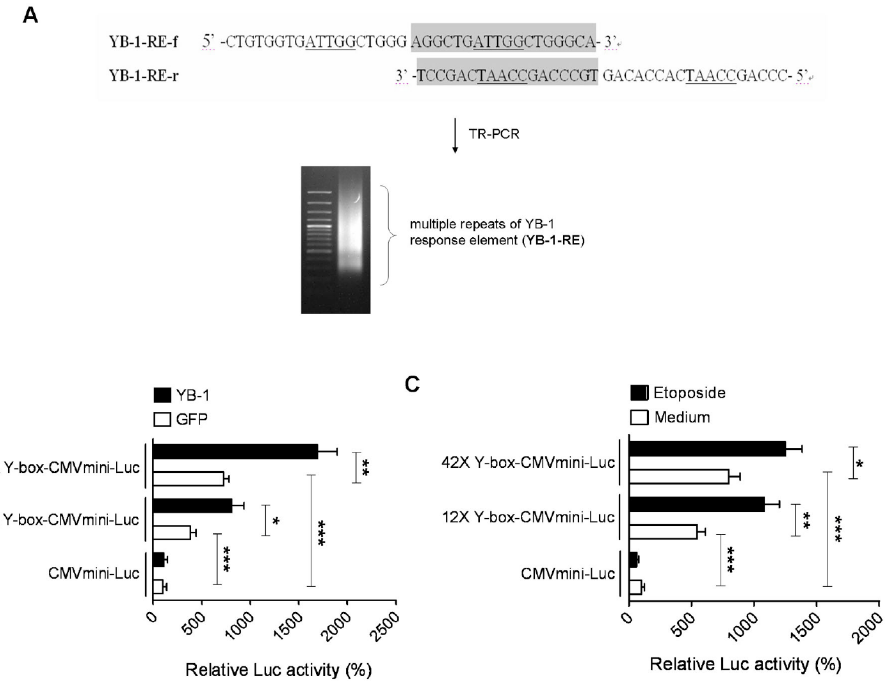

Supplementary Figure S3: Construction of multiple copies of the YB-1 responsive element (Y-box). Multiple copies of the Y-box were obtained by template repeated-PCR. The oligonucleotides YB-1-RE-f (5'-CTGTGGTGATTGGCTGGGAGGCTGATTGGCT GGGCA-3') and YB-1-RE-r (5'-CCCAGCCAATCACCACAGTGCCCAGCCAAT CAGCCT-3') were used as primers and templates in the reaction. **A.** Oligonucleotide primers for TR-PCR. The sequence highlighted in grey (from −84 to −68 of the human *MDR1* promoter region) within YB-1-RE-f is complementary to that of YB-1-RE-r. Underlines indicate the Y-box. The PCR products were separated in 1% agarose gel electrophoresis. The resulting DNA fragments containing multiple copies of the Y-box were cloned into a TA cloning vector and subsequently sequenced to verify their correctness. The 12 and 42 copies of the Y-box were obtained and then subcloned into pGL3-CMVmini-Luc to generate pGL3-12×Y-box-CMVmini and pGL3-42×Y-box-CMVmini, respectively. **B.** Analysis of the responsiveness of the Y-box by overexpression of YB-1. A549 cells were cotransfected with pGL3-CMVmini-Luc, pGL3-12×Y-box-CMVmini, or pGL3-42×Y-box-CMVmini together with pLKO.1-puro-HA-YB-1 or a control vector (pLKO.1-puro-GFP). After 48 h, luciferase activities of the cells were determined. **C.** Etoposide enhances the Y-box responsiveness in A549 cells. The cells were transfected with pGL3-CMVmini-Luc, pGL3-12×Y-box-CMVmini, or pGL3-42×Y-box-CMVmini. After 24 h, cells were exposed to various concentrations of etoposide or epirubicin for an additional 24 h. Luciferase activities of the cells were determined.*, $P < 0.05$; **, $P < 0.01$; ***, $P < 0.001$.

Supplementary Table S1: Genes upregulated by Ad5WS1 in MCF-7 cells.
