# Supplementary material for: Etoposide enhances antitumor efficacy of MDR1-driven oncolytic adenovirus through autoupregulation of the MDR1 promoter activity
Source: Oncotarget. 2015 Oct 16;6(35):38308–26. doi: 10.18632/oncotarget.5702 (PMC4742001; doi:10.18632/oncotarget.5702)
Supplement: Supplementary file 2 [file oncotarget-06-38308-s002.doc]

**Supplementary table**

**
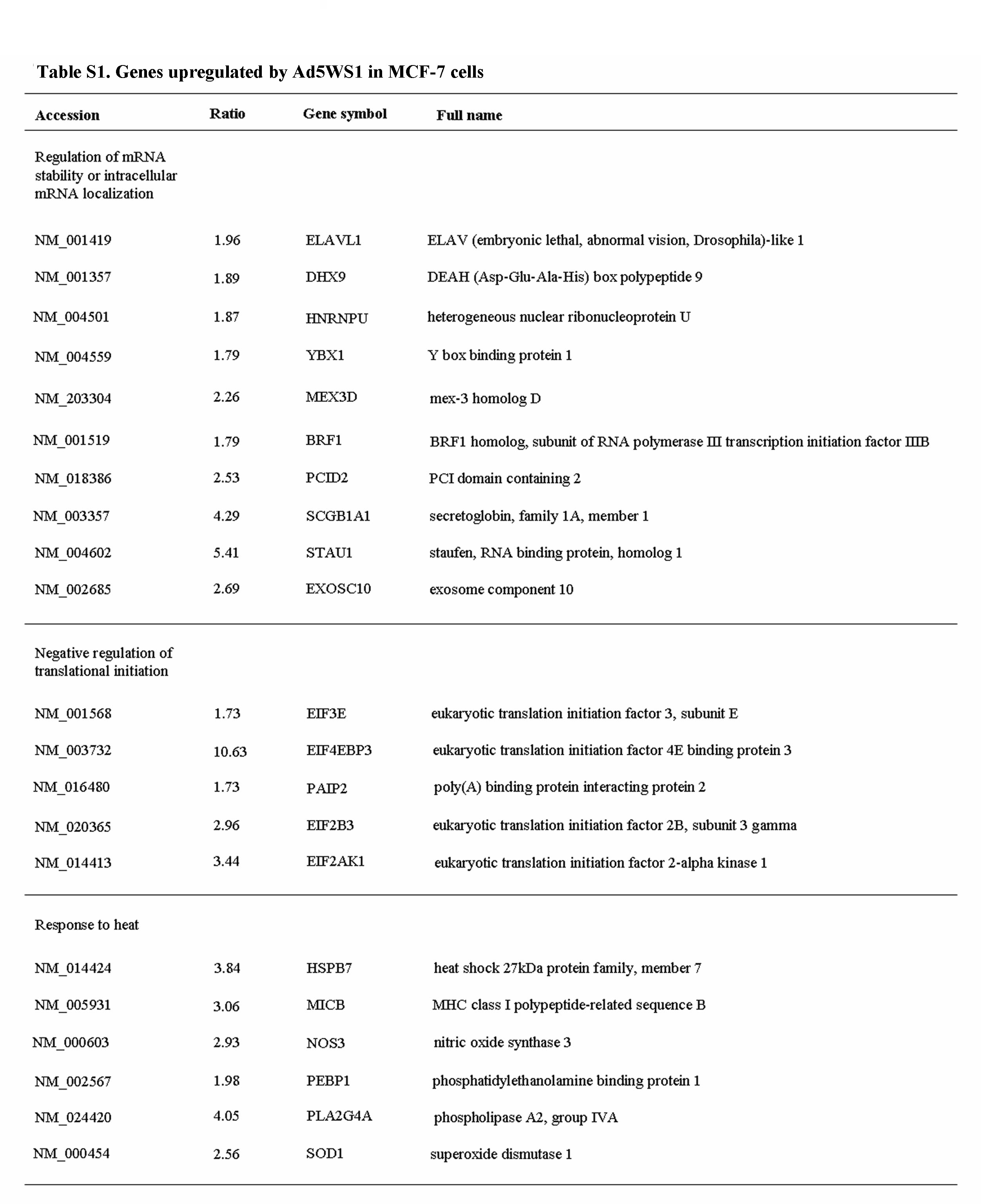
**

Total RNA was extracted from MCF-7 cells infected with Ad5WS1 or Adnull, and their cDNA was synthesized and labeled with Cy5. Microarray hybridization was carried out using Phalanx Biotech Spotted Microarray (HOA_005.1).
